# Comparative assessment of autologous and allogeneic iNKT cell transfer in iNKT cell-based immunotherapy

**DOI:** 10.3389/fimmu.2024.1457771

**Published:** 2024-08-19

**Authors:** Mariko Takami, Shinichiro Motohashi

**Affiliations:** Department of Medical Immunology, Graduate School of Medicine, Chiba University, Chiba, Japan

**Keywords:** invariant natural killer T cells, adoptive immunotherapy, cancer immunotherapy, allogeneic cells, induced pluripotent stem cells

## Abstract

Invariant natural killer T (iNKT) cells are a small subset of T lymphocytes that release large amounts of cytokines such as IFN-γ and exhibit cytotoxic activity upon activation, inducing strong anti-tumor effects. Harnessing the anti-tumor properties of iNKT cells, iNKT cell-based immunotherapy has been developed to treat cancer patients. In one of the iNKT cell-based immunotherapies, two approaches are utilized, namely, active immunotherapy or adoptive immunotherapy, the latter involving the *ex vivo* expansion and subsequent administration of iNKT cells. There are two sources of iNKT cells for adoptive transfer, autologous and allogeneic, each with its own advantages and disadvantages. Here, we assess clinical trials conducted over the last decade that have utilized iNKT cell adoptive transfer as iNKT cell-based immunotherapy, categorizing them into two groups based on the use of autologous iNKT cells or allogeneic iNKT cells.

## 1 Introduction

Invariant natural killer T (iNKT) cells were identified in the late 1980s as a distinct population of T cells expressing both T-cell receptor (TCR) and NK cell markers (1–3). iNKT cells express invariant TCRs, consisting of a Vα24-Jα18 chain and Vβ11 chain in humans, and a Vα14-Jα18 chain and Vβ8.2, Vβ7, or Vβ2 chain in mice. These TCRs recognize their cognate glycolipid presented on the MHC class I-like molecule CD1d, and can produce large amounts of cytokines including IFN-γ. Meanwhile, iNKT cells directly exert tumoricidal activity upon activation (4, 5). The discovery of iNKT cells’ potent immunomodulatory effects sparked interest in their therapeutic potential. Furthermore, the development of iNKT cell-based immunotherapy was accelerated upon the discovery of the cognate ligand α-galactosylceramide (α-GalCer) (2).

Clinical trials have been initiated to evaluate the safety and efficacy of iNKT cell-targeted therapies in humans. About 20 years ago, we embarked on a phase I clinical study of iNKT cell-based immunotherapy targeting non-small cell lung cancer (NSCLC) patients (6). In that study, autologous α-GalCer-pulsed antigen-presenting cells (APCs) were generated *ex vivo* and infused into patients, with the expectation that they would activate endogenous iNKT cells, which would subsequently trigger other anti-tumor immune responses in patients to fight cancer. This work showed promising results, so other clinical trials using similar methods targeting NSCLCs and head and neck cancers (HNCs) were conducted at our facility (7–12). In 2020, the results of a single-arm phase II clinical trial of α-GalCer-pulsed APCs as a second-line treatment for advanced or recurrent NSCLC was reported (13). The intravenous injection of α-GalCer-pulsed APCs was well-tolerated and was accompanied by prolonged overall survival. The median overall survival time of all 35 enrolled patients was 21.9 months, which was better than the expected survival time. However, we encountered challenges such as limited clinical efficacy due to a lower percentage of iNKT cells in cancer patients than in healthy donors (14–16). To begin with, a certain number of autologous iNKT cells have to be present in the body in the case of active immunotherapy. In adoptive immunotherapy, iNKT cells can be expanded *ex vivo* before infusion into patients, increasing the likelihood of inducing iNKT cell responses upon activation. In adoptive transfer therapy, there are two types of iNKT cells that can be infused: autologous or allogeneic. While the adoptive transfer of *ex vivo* expanded iNKT cells was not effective as transferring α-GalCer-pulsed APCs in our past clinical trials, adoptive transfer of iNKT cell therapy is gaining attention with the advancement of combination therapies and novel techniques such as transducing chimeric antigen receptor (CAR). In this review, we discuss the advantages and disadvantages of adoptive iNKT cell-based immunotherapy using autologous or allogeneic iNKT cells.

## 2 Autologous iNKT cell transfer

The main advantage of using autologous iNKT cells for adoptive transfer therapy is the avoidance of rejection via host immune responses; thus, transferred iNKT cells are expected to persist longer than allogeneic iNKT cells. In addition, autologous iNKT therapy allows us to establish personalized treatment approaches tailored to individual patients’ characteristics, including HLA type and immune status. Moreover, autologous iNKT therapy avoids the risk of infection from donor cells. However, there are disadvantages including difficulty obtaining a sufficient number of functional autologous iNKT cells, and the fact that the procedure is time-consuming and expensive because of the need to manufacture cell products individually (**Table 1**).

**Table 1:** Comparison of autologous and allogeneic iNKT cells.

Origin of iNKT cells	Advantages	Disadvantages
Autologous	• Avoidance of rejection • Avoidance of infection from donor cells • Tailored to individuals	• Time consuming • Expensive
Allogeneic	• Easy to handle • Enables off-the-shelf therapy • Avoidance of mmune exhaustion	• Shorter persistence • Risk of infection from donor cells

### 2.1 Expansion of autologous iNKT cells

We performed a phase I clinical trial of autologous iNKT cell infusion targeting recurrent or advanced NSCLC patients (n=6) (9). iNKT cells were expanded from peripheral blood mononuclear cells (PBMCs) of patients in the presence of α-GalCer and IL-2 *ex vivo*. A bulk population of cultured cells including iNKT cells (0.3% to 21.5%) was then infused into the patients intravenously. No adverse events were observed in this study. Adverse event was defined based on common terminology criteria for adverse events (CTCAE, https://ctep.cancer.gov/protocolDevelopment/electronic_applications/ctc.htm). In general, adverse events are classified by their severity from Grade 1 (mild) to Grade 5 (death). Furthermore, some of the patients showed increased proportions of iNKT cells and IFN-γ-producing cells among PBMCs, indicating the immune responses after infusion of the iNKT cells. Based on these promising findings, we also conducted phase I and phase II clinical trials, investigating adoptive transfer of both iNKT cells and α-GalCer-pulsed APCs for HNC patients (n=18) (11, 12). iNKT cells were injected into a tumor-feeding artery. This combined therapy was associated with a serious adverse event in 1 patient (phase I) with a pharyngo-cutaneous fistula related to local tumor reduction. The remaining 17 patients experienced only mild adverse events and 8 patients (3 patients in phase I and 5 patients in phase II) achieved partial responses, suggesting the enhancement of anti-tumor immune responses.

There are several ways of expanding iNKT cells and the proportion of iNKT cells also differs among individuals. Exley et al. expanded and generated iNKT cells at much higher purity for adoptive therapy. In a phase I clinical study, the expanded autologous iNKT cells with much higher purity (13%–87%) were used for adoptive transfer into advanced melanoma patients (n=9) (17). In the study, iNKT cells were isolated using a monoclonal antibody (6B11) against the invariant TCRα chain expressed by these cells, followed by expansion using anti-CD3 antibody in the presence of IL-2. Grade 1–2 toxicities were observed, while an increased number of iNKT cells were detected after infusion.

### 2.2 Global clinical trials of iNKT cell-based immunotherapy

In the last 10 years, various clinical studies have been performed on adoptive autologous iNKT cell therapy globally. Gao et al. reported on the adoptive transfer of autologous iNKT cells (85%–95%) targeting advanced hepatocellular carcinoma (n=10) (18). In that study, PBMCs were stimulated with α-GalCer in the presence of IL-2 for the first round of expansion, followed by the sorting of iNKT cells using magnetic beads. Then, purified iNKT cells were co-cultured with autologous mature DCs as the second round of expansion. Grade 1–2 toxicities were observed in most of the patients, while grade 3 adverse events were reported in three patients, suggesting that infusion of autologous iNKT cells was safe and well tolerated. The latest study by the same group was a phase II clinical trial (NCT04011033, n=54) investigating the combination therapy of autologous iNKT cell infusion with trans arterial embolization for hepatocellular carcinoma (19). Here, patients were randomly assigned to either trans arterial embolization alone or in combination with iNKT cell infusion. For the combination treatment, expanded iNKT cells (purity >95%) were infused into patients along with trans arterial embolization treatment. The results indicated that the infusion of iNKT cells significantly improved progression-free survival, overall response rate, disease control, and quality of life while keeping toxicity at manageable levels. Additionally, other combination therapies involving iNKT cell infusion have been reported. For example, a phase I/II clinical trial of combination therapy of autologous iNKT cells and PD-1^+^CD8^+^ T-cell infusion targeting advanced NSCLC patients was conducted (NCT03093688, 2017–2022) (20). Autologous iNKT cells were expanded from PBMCs with the stimulation of α-GalCer in the presence of IL-2 and IL-7, followed by the addition of α-GalCer-pulsed DCs. Autologous PD1^+^CD8^+^ T cells sorted from PBMCs were expanded using anti-CD3/anti-CD28-coated beads in the presence of IL-2, IL7, IL-15, and TLR agonists. Three patients were enrolled in this study and autologous iNKT cells (purity 13% to 88%) and PD-1^+^CD8^+^ T cells (purity >95%) were infused into them for multiple cycles (6, 10, or 16 cycles). In that study, the infusion of iNKT cells and PD-1^+^CD8^+^ T cells induced grade 1–2 toxicities, but the patients tolerated it well. The same group has also conducted the combination therapy of autologous iNKT cells and PD-1^+^CD8^+^ T-cell infusion for patients with advanced pancreatic cancer (NCT03093688) (21). In that study, nine patients received at least three cycles of cell infusion, with no adverse events observed. Furthermore, overall survival of 5 patients were over 15 month and showed a potentially promising prolonged overall survival time compared to patients who received general chemo therapies (22).

### 2.3 Utilizing autologous iNKT cells for CAR-NKT cell therapy

Chimeric antigen receptor T-cell (CAR T-cell) therapy is one of the major immunotherapeutic options for certain cancer patients and has shown remarkable success for hematological malignancies. CAR consists of an extracellular antigen recognition domain derived from a monoclonal antibody, which recognizes a specific antigen expressed on cancer cells. CAR-NKT cell therapy has been developed and a phase I/II clinical trial of therapy with GD2 CAR NKT cells expressing IL-15 has been performed (NCT03294954) (23, 24). NKT cells with median purity of 93% were retrovirally transduced with GD2 CAR and IL-15 with median 60.1% efficiency. While this study is ongoing, preliminary results indicate a 25% response rate among neuroblastoma patients (n=12) who received this therapy, with one patient demonstrating a complete response, affirming the effective trafficking of CAR-NKT cells to the tumor site.

Other clinical trials of autologous NKT cell infusion targeting NSCLC, renal cell carcinoma, and melanoma have been registered at ClinicalTrials.gov, although no results have been reported yet (e.g., NCT02562963, NCT03198923, NCT06182735, NCT02619058).

## 3 Allogeneic iNKT cell transfer

In recent years, adoptive transfer of allogeneic iNKT cells has been developed as a new therapeutic tool to treat cancer patients in parallel with autologous iNKT cell therapy. There are at least three advantages of using allogeneic iNKT cells compared with autologous iNKT cells in cell therapy (**Table 1**). First, the use of allogeneic iNKT cells enables the development of off-the-shelf therapy. Off-the-shelf therapy can reduce the cost and streamlines the process, shortening the time from cell preparation to shipment, since there is no need to customize or individualize the preparation of cells. Second, the use of allogeneic iNKT cells allows the simple preparation of a sufficient number of iNKT cells. It has been reported that the proportion of iNKT cells in the peripheral blood of cancer patients is decreased compared with that in healthy donors (14–16), so obtaining enough autologous iNKT cells for treatment has often been challenging. Meanwhile, the expansion of iNKT cells derived from healthy donors is relatively easy. Moreover, cord blood, which is comparatively accessible and contains a large number of T cells, can be used as a source of allogeneic iNKT cells. Third, allogeneic iNKT cells derived from healthy donors enable the avoidance of immune exhaustion. T cells in the tumor microenvironment are exhausted, as characterized by the expression of PD-1, TIM-3, and LAG-3, while iNKT cells derived from a healthy donor are expected to be less so (25). In addition, TCR expressed on iNKT cells, unlike TCR expressed on conventional T cells, does not recognize antigens in an MHC-restricted manner, which is a significant advantage as it reduces the risk of developing graft-versus-host disease (GVHD).

Meanwhile, a potential disadvantage of using allogeneic iNKT cells is shorter persistence, compared with that of autologous iNKT cells after infusion into the host, because of host immune responses of allogeneic reaction.

### 3.1 Utilizing allogeneic iNKT cells from healthy donors

MINK Therapeutics developed the protocol of allogeneic iNKT cell expansion for off-the-shelf therapy to use in clinical trials (26). Briefly, iNKT cells isolated from healthy donors were expanded under stimulation with α-GalCer in the presence of cytokines. The function of these cells was confirmed by cytotoxicity assay and cytokine production against a tumor cell line. Unmodified allogeneic iNKT cells derived from healthy donors were stimulated with α-GalCer in the presence of cytokines for expansion and adoptively transferred to 34 solid tumor patients, including those with NSCLC, pancreatic, rectal, cholangiocarcinoma/biliary duct, and other cancers (NCT05108623) (27). Whereas allogeneic iNKT cells were expected to be eliminated via an allogeneic reaction by host immune cells, peripheral allogeneic iNKT cells were detected at a consistent level up to 8 weeks after the adoptive transfer. The combination of allogeneic iNKT cell therapy with pembrolizumab or nivolumab, both of which are immune check point inhibitors targeting programmed cell death 1 receptor (PD-1), was also examined in six patients, along with monotherapy of allogeneic iNKT cell transfer in 26 patients (28). The study suggested that both this monotherapy and these two combination therapies were well tolerated. Notably, one patient with gastric cancer who was resistant to nivolumab treatment showed a partial response, while increased immune infiltration, activation, and Th-1 polarization were observed after iNKT cell infusion (29).

Other combination therapies with allogeneic iNKT cell transfer have also been performed in China. Yu et al. reported on a phase I/II clinical trial targeting non-small-cell lung cancer patients. NSCLC patients bearing epidermal growth factor receptor (EGFR) mutation received allogeneic iNKT cell infusion along with gefitinib, an EGFR inhibitor (30). Thirty patients were randomly assigned to receive either monotherapy of gefitinib or combination therapy of gefitinib with allogeneic iNKT cell infusion. One EGFR mutation-positive NSCLC patient who received this combination therapy showed a delay in the development of molecular-targeted drug resistance with no adverse events (31).

iNKT cells play an important role not only for fighting tumor cells but also for clearing bacterial and viral infections. A phase I/II clinical trial of allogeneic iNKT cell therapy for treating acute respiratory distress syndrome (ARDS) patients secondary to SARS-CoV-2 infection, has been performed (n=20) (NCT04582201) (32). That study showed the safety of treatment without toxicities and the persistence of allogeneic iNKT cells in patients infused with iNKT cells. They also showed that injected allogeneic iNKT cells rescued exhausted T cells and killed M2 macrophages promoting infection. Infused iNKT cells induced an anti-inflammatory systemic response, which contributed to the stabilization of ARDS.

In addition to combination therapy, CAR therapy using allogeneic iNKT cells has been investigated. Ramos et al. performed clinical studies to examine allogeneic iNKT cells transduced with CD19 CAR along with shRNA targeting β2 microglobulin and CD74, which downregulate HLA class I and class II and thereby avoid elimination by host immune responses via an allogeneic reaction. Seven patients with relapsed/refractory B-cell non-Hodgkin lymphoma (NHL) and two patients with relapsed B-cell lymphoblastic leukemia (ALL) were enrolled (NCT00840853) (33). In that study, two NHL patients and one ALL patient exhibited complete responses, suggesting that allogeneic CAR iNKT cells induce strong anti-tumor immunity.

### 3.2 Utilizing allogeneic iPS cell-derived iNKT cells

Induced pluripotent stem cells (iPSCs) are reprogrammed adult cells that regain pluripotency, allowing them to differentiate into various cell types, including immune cells. Yamada et al. established techniques to generate iPSC-NKT cells from adult iNKT cells and confirmed that iPSC-NKT cells possess anti-tumor activity matching that of the primary iNKT cells (34). The iPSC technique offers a significant advantage by enabling the mass expansion of a limited number of iNKT cells. Based on these results, we are currently conducting the first-in-human clinical trial using allogeneic iPSC-iNKT cells as a monotherapy for treating head and neck cancer patients (jRCT2033200116). iPSC-NKT cells derived from HLA-mismatched donors were infused into patients with advanced HNC to examine the safety and clinical efficacy of this treatment. The safety of administrating high doses of iPSC-NKT cells has been confirmed so far.

## 4 Discussion

iNKT cell-based immunotherapy has advanced dramatically over the last 20 years, with new clinical trials, including combination therapies, continually emerging. The use of allogeneic iNKT cells for such therapy was a particular breakthrough, overcoming the key weakness of iNKT cells of a low cell number and further accelerating clinical trials with greater potential. Both autologous and allogeneic iNKT cell transfer have advantages and advantages as listed in **Table 1**. In all of the above-mentioned clinical trials on the adoptive transfer of either autologous and allogeneic iNKT cells, safety and a certain level of immunological responses with some clinical efficacy were confirmed, suggesting the promise of these treatment options for cancer patients (**Table 2**). In a pre-clinical study using murine models, Li et al. reported that allogeneic CAR-iNKT cells derived from hematopoietic stem cells became resistant to host cell-mediated allorejection by editing the HLA gene (35). They recently improved the CAR-iNKT cell culture method using feeder free system and brought the technology closer to clinical application (36). In addition, Heczey et al. showed that knocking down BTG proliferation factor 1 (BTG1) rescued the hyporesponsiveness of CAR-NKT cells (24). These findings can be applied for the next step of new iNKT-cell-based therapy to enhance the anti-tumor function and persistence of iNKT cells. These gene editing techniques hold the potential to significantly enhance the efficacy of iNKT cell-based immunotherapy, thereby improving outcomes in the fight against various types of cancers.

**Table 2:** Clinical trials of iNKT cell adoptive transfer.

References	Source	Expansion methods from PBMCs	Purity of iNKT cells (%)	Type of cancer	Number of patients	Phase	Combination
(9)	Autologous	▾expansion (+ α-GalCer, IL-2)	0.1-25	NSCLC	6	I	
(10)			1-59	HNC	8	I	α-GalCer-APCs
(11)			0.1-6.2	HNC	10	II	α-GalCer-APCs
(17)		▾sorting iNKT cells ▾expansion (+anti-CD3, IL-2)	13-87	Melanoma	9	I	
(18)		▾expansion (+α-GalCer, IL-2) ▾sorting iNKT cells ▾coculture (+autologous DCs)	85-95	HCC	10	I	
(19)			N/A	HCC	27 27 (Ctrl)	II	Trans-arterial embolization
(20)		▾expansion (+α-GalCer) ▾+IL-2, IL-7 ▾coculture (+autologous DCs) ▾+IL-12 ▾+IL-15	13-88	NSCLC	3	I/II	PD1^+^CD8^+^ T cells
(21)			5-65	PC	9	I	PD1^+^CD8^+^ T cells
(23, 24)		▾sorting iNKT cells ▾expansion (+irradiated PBMCs, α-GalCer, IL-2, IL-21) ▾transducing GD2-CAR-IL-15	74-97	NB	12	I	
(27)	Allogeneic	▾sorting iNKT cells ▾expansion (+irradiated PBMCs, α-GalCer, IL-2)	>99	Solid tumor	34	I	
(28)			>99	Solid tumor	26 6 (Combo)	I	Pembrolizumab or Nivolumab
(30)		N/A	>20	NSCLC	15 15 (Ctrl)	I/II	Gefitinib
(33)		▾transducing CD19-CAR-IL-15, HLA ablation	N/A	NHL, ALL	9	I	
N/A		▾expansion (+α-GalCer, IL-2) ▾expansion (+mouse DCs, IL-2, IL-7, IL-15) ▾inducing iPS cell-derived NKT cells	>80	HNC	10	I	

NSCLC, non-small cell lung cancer; HNC, head and neck cancer; PC, pancreatic cancer; NB, neuroblastoma; NHL, non-Hodgkin lymphoma; ALL, acute lymphoblastic leukemia; N/A, not available; iPS, induced pluripotent stem; Ctrl, control.
